# Geographic Stomatitis with Multiple Areas Involvement of Oral Cavity: A Case Report and Review of Literatures

**DOI:** 10.31661/gmj.v10i0.2107

**Published:** 2021-08-09

**Authors:** Farid Abbasi, Aliyeh Sehatpour, Seyed Masoud Sajedi, Parisa Bahadori, Mohadeseh Nouri

**Affiliations:** ^1^Oral Medicine Department, Faculty of Dentistry, Shahed University, Tehran, Iran

**Keywords:** Geographic Stomatitis;, Geographic Tongue;, Oral Mucosa

## Abstract

**Background:**

Geographic stomatitis is an uncommon migratory benign lesion of oral mucosa with unknown etiology. It can affect all the areas of the oral mucosa, but the dorsum and lateral borders of the tongue are the most commonly involved areas called geographic tongue. The clinical appearance of this condition is the oval or circular erythematous areas with irregular elevated keratotic borders. These characteristic features of geographic stomatitis are diagnostic for all clinicians when appearing on the dorsum of the tongue, despite other affected areas of oral mucosa that can confuse clinicians. This condition may be associated with some diseases such as psoriasis, Reiter’s syndrome, and atopic conditions, so the clinicians should rule out these diseases and diagnose the geographic stomatitis.

**Case Report:**

A 17-year-old male attended to our department for a routine dentistry checkup. During the intraoral examination, we found multiple erythematous areas surrounded by a thin white border on different surfaces of his oral cavity. His extraoral examinations were completely normal.

**Conclusion:**

Due to the rarity of this lesion on the other sites of oral mucosa rather than dorsum and lateral borders of the tongue such as labial mucosa, buccal mucosa, the floor of the mouth, ventral surface of the tongue, and palate, it is necessary to report, study and evaluate each case of this condition that clinicopathologic findings have confirmed this diagnosis, to treat and advice these patients on the best approach.

## Introduction

Geographic stomatitis or migratory stomatitis is an uncommon benign condition of unknown etiology reported by Cook in 1955 [1]. Synonyms describing the clinical appearance of this condition include erythema migrans, annulus migrans, migratory mucositis, and ectopic geographic tongue [1,2,3]. In the clinical examination, geographic stomatitis is typically characterized by single or multiple circular erythematous areas surrounded by white, yellow, or gray changes in the periphery. The location of these lesions can differ from point to point in the oral mucosa [4]. The tongue's dorsal aspect and lateral borders are the most common locations of this condition called geographic tongue [5]. Its prevalence is about 1-2% of the general population [6]. Histopathologic features of geographic stomatitis and tongue include regulated elongated rete ridges, elongated and edematous papillae, parakeratoses, and Munro’s microabscesses [7]. Symptoms of this mucosa disorder are rarely reported, and a burning sensation aggravating during stress periods is the most common complaint via patients with symptomatic geographic stomatitis [4]. According to the clinical distribution of geographic stomatitis, there is HUME classification which is described as below [7]: Type 1: Lesions involved only dorsum and lateral borders of the tongue. This type only has a geographic tongue without any lesion on the other area of the oral mucosa. Type 2: In addition to tongue involvement, there are lesions on the other surfaces of the oral mucosa. It means the geographic tongue is accompanied by geographic stomatitis. Type 3: Atypical or fixed geographic lesions that can accompany lesions on the other sites of the oral mucosa. Indeed, atypical geographic tongue with or without geographic stomatitis. Type 4: Any oral mucosa site can be affected by this condition except the dorsum and lateral borders of the tongue. Actually, we have only geographic stomatitis without any tongue involvement. This report presented a young man with multiple oral cavity areas involvement without any acute symptoms.

## Case Presentation

**Figure 1:**
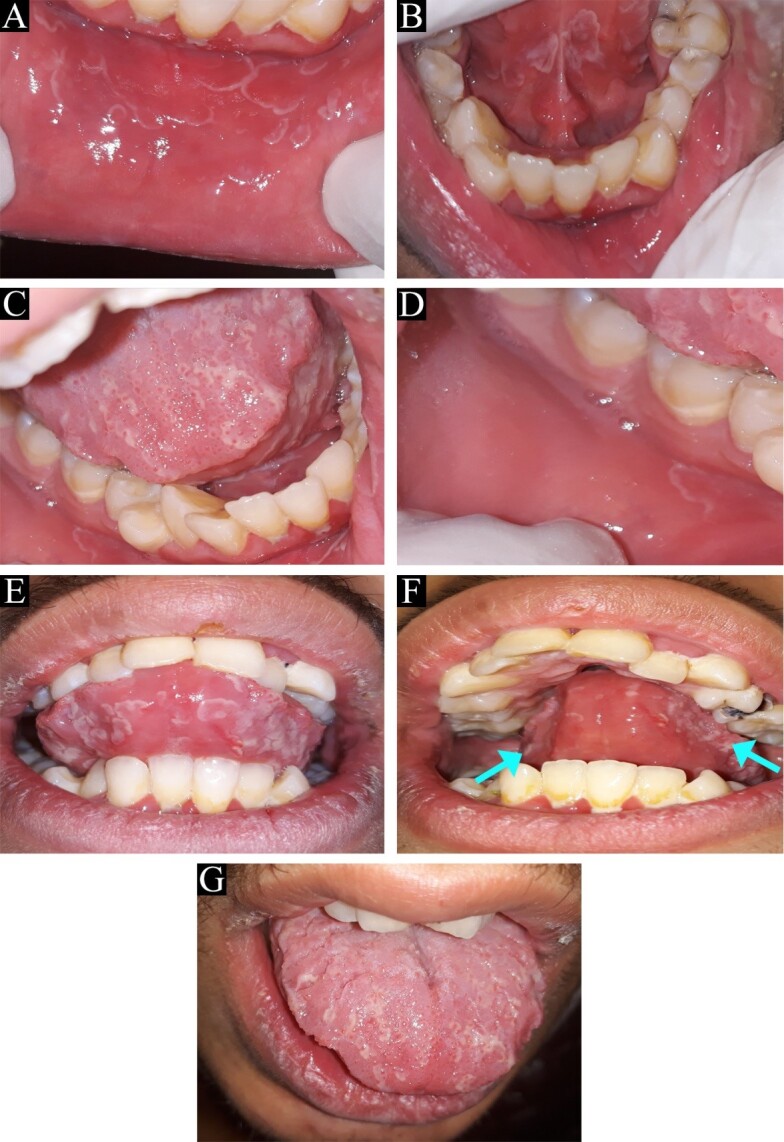
Geographic stomatitis on the (A) labial mucosa, (B) & (C) floor of the mouth, (D) buccal mucosa, (E) ventral surface of the tongue, (F) lateral borders of the tongue, (G) dorsal surface of the tongue.

**Figure 2:**
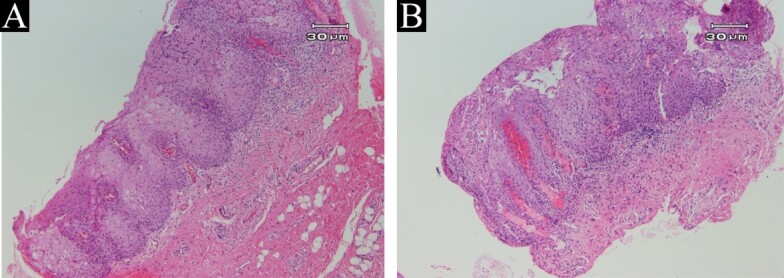
Histopathologic figure of the (A) ventral surface of the tongue, (B) labial mucosa.

**Figure 3:**
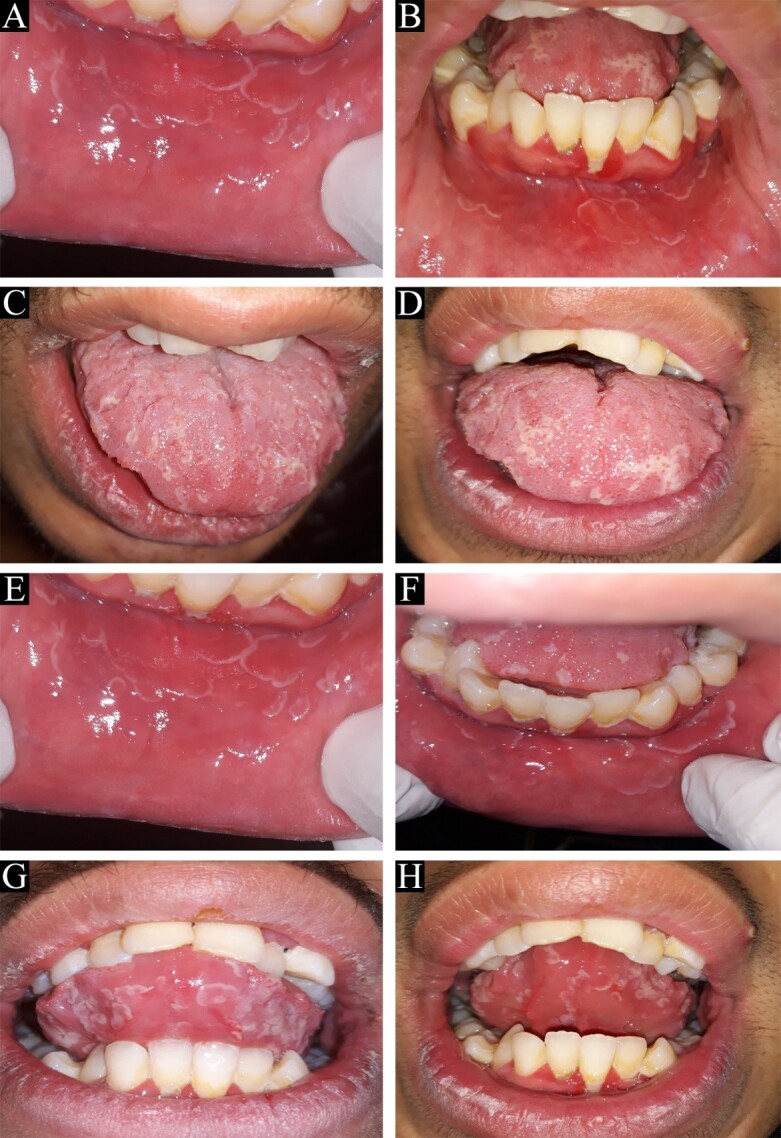
Labial mucosa (A) at first visit & (B) in 3 months follow up, Dorsal surface of the tongue (C) at first visit & (D) in 3 months follow up, Labial mucosa (E) at first visit & (F) in 6 months follow up, ventral surface of the tongue (G) at first visit & (H)in 6 months follow up.

A 17-year-old male applied to the Oral and Maxillofacial Department of School of Dentistry at Shahed Universit for routine oral and dental checkups. Intraoral clinical examination revealed multiple erythematous areas surrounded by a thin white border with a slightly elevated appearance on the labial mucosa (Figure-1A), the floor of the mouth (Figure-1B and C), buccal mucosa (Figure-1D), ventral surface of the tongue (Figure-1E), lateral borders of the tongue (Figure-1F), and dorsum of the tongue (Figure-1G). In addition to these lesions, the fissured tongue was another abnormal appearance of oral mucosa in this patient. All of the lesions were asymptomatic, and the patient was not aware of their presence. The extraoral examination (e.g., skin, eyes, joints) was completely normal. Also, the medical and familial history was not relevant. Often these clinical features of the tongue are characteristic and enough to diagnose. However, due to the rarity of involvement of other sites of the oral mucosa, (such as labial and buccal mucosa, floor of the mouth, and ventral surface of the tongue), in this case, a histopathologic examination was necessary to confirm the diagnosis. Therefore, the biopsy was considered. After explaining the condition to the patient, he filled a consent form to perform biopsies and published the results in the article. Then two biopsy samples were kept from the ventral surface of the tongue and labial mucosa, respectively. Histopathologic findings confirmed the diagnosis of geographic stomatitis (Figure-2). Microscopic description of these two samples revealed stratified squamous mucosa with moderate acanthosis and regular elongation of rete pegs, thinning of suprapapillary plates, and prominent exocytosis of neutrophilic inflammatory cells producing intraepithelial superficial spongiform pustules (Figure-2). There is also prominent infiltration of neutrophilic inflammatory cells in surface parakeratotic layers. The underlying subepithelial corium with moderate dense infiltrates of mixed inflammatory cells. The special Periodic acid–Schiff staining– staining method to detect polysaccharides such as glycogen, and mucosubstances such as glycoproteins, glycolipids, and mucins in tissues–failed to reveal fungal elements. Due to the clinical examination and histopathologic description, the diagnosis of geographic stomatitis was confirmed as the etiology of this condition is unknown; there is no etiological strategy for treatment. Thus, we informed the patient about the disorder’s benign character and asked him to refer us when he feels symptoms, e.g., burning sensation. The patient followed up for six months. As expected, at three and six months of follow-up, the displacement of the lesions due to the nature of the geographic stomatitis was quite evident (Figure-3), and the patient had no symptoms during this time, including burning or pain.

## Discussion

Geographic stomatitis, known by other names such as migratory stomatitis, ectopic geographic tongue, Cooke’s disease, or migratory mucositis is often asymptomatic, and its distribution is equal in men and women [1,4,5]. Due to being asymptomatic of these lesions in most patients and lack of awareness of presenting these lesions in the oral cavity, determining the exact prevalence of this condition is complicated [8]. However, its prevalence is predicted at about 1-2% of the general population [6,9]. The most common sites of geographic stomatitis are the tongue, then buccal mucosa, lower labial mucosa, muccobuccal fold, vestibule, the floor of the mouth, lips, and soft palate, respectively [10]. Our patient has lesions on the dorsal and lateral borders of the tongue, buccal mucosa, labial mucosa, ventral surface of the tongue and, the floor of the mouth. According to HUME classification, our patient is categorized in type 2 lesions [7]. The etiology of geographic stomatitis is not entirely understood [10]. The hereditary pattern of this condition in some families introduces genetics as a probable etiologic or predisposing factor [4]. In our patient, none of the family members had geographic stomatitis or geographic tongue. Geographic stomatitis can be related to psoriasis as an immunity disorder. Some researchers suggest that geographic stomatitis is an oral manifestation of psoriasis [7,11,12,13]. Our patient did not have any cutaneous signs and symptoms. There is a possible link between geographic stomatitis and Reiter’s syndrome [3,4,7,10]. This syndrome is characterized by arthritis, uveitis or conjunctivitis, and urethritis [4]. This association was rule out in our case based on ophthalmologic, rheumatologic, urologic, and genetic examinations. Geographic tongue is a recurring migrating condition. The erythematous areas of this condition are the result of the atrophy of the filiform papillae. After some time, when the healing process of depapillated erythematous areas begun, the peripheral keratotic areas disappear, and another site presents this red and white condition. This process that has been mentioned explains the migratory nature of this lesion. However, based on the activity of the lesions, one or more areas may be involved at the same time [4]. All the affected areas (dorsal, ventral, and lateral borders of the tongue, the floor of the mouth, labial mucosa, and buccal mucosa) present multiple locations of involvement with a migratory pattern at our patient different times. Our first differential diagnosis was confirmed based on the agreement of histopathological findings from our case samples with the histopathological features as expressed by Lever in 1975 [7]. Our differential diagnoses were geographic stomatitis, Lichen planus, lupus erythematous, and oral candidiasis. The clinical appearance of the lesions was not adopted to other differential diagnoses. Due to the unknown etiology of this disorder, therapeutic strategies are based on the severity and presence of symptoms. In asymptomatic patients, such as our case, informing the patient about this lesion's benign nature is sufficient [4]. While in symptomatic patients, the severity of the symptoms has a crucial role in drug prescription. Topical analgesic agents, antihistamines, anxiolytic drugs, and steroids are drugs of choice that can be prescribed [4,5,10]. Vitamin A and topical tacrolimus are suggested as effective drugs for this lesion [5,10].

## Conclusion

Geographic stomatitis as erythematous areas of all the surfaces of oral mucosa is a benign condition that modifies oral epithelium and connective tissue that might or not be relevant to diseases such as psoriasis and Reiter’s syndrome. The palliative and symptomatic treatments are the only therapeutic strategies for this condition.

## Acknowledgment

The authors thank the Faculty of Dentistry of Shahed University.

## Conflict of Interest

All authors declare no conflict of interests related to this study.
